# Supplementary material for: Modeling glioblastoma heterogeneity as a dynamic network of cell states
Source: Mol Syst Biol. 2021 Sep 16;17(9):e10105. doi: 10.15252/msb.202010105 (PMC8444284; doi:10.15252/msb.202010105)
Supplement: Supplementary file 5 — Source Data for Figure 3 [file MSB-17-e10105-s001.zip › Figure3A_sourcedata/GSEA_3065/hallmarks_state2.GseaPreranked.1623416269049/HALLMARK_MYC_TARGETS_V2.html]

Details for gene set HALLMARK\_MYC\_TARGETS\_V2[GSEA]

|  || Dataset | state2 |
| Phenotype | NoPhenotypeAvailable |
| Upregulated in class | na\_pos |
| GeneSet | HALLMARK\_MYC\_TARGETS\_V2 |
| Enrichment Score (ES) | 0.6709732 |
| Normalized Enrichment Score (NES) | 2.0182145 |
| Nominal p-value | 0.0 |
| FDR q-value | 0.0 |
| FWER p-Value | 0.0 |
Table: GSEA Results Summary

  

Fig 1: Enrichment plot: HALLMARK\_MYC\_TARGETS\_V2      
 Profile of the Running ES Score & Positions of GeneSet Members on the Rank Ordered List

  

| PROBE | GENE SYMBOL | GENE\_TITLE | RANK IN GENE LIST | RANK METRIC SCORE | RUNNING ES | CORE ENRICHMENT || 1 | MCM4 |  |  | 79 | 0.399 | 0.0616 | Yes |
| 2 | MCM5 |  |  | 83 | 0.394 | 0.1305 | Yes |
| 3 | CDK4 |  |  | 131 | 0.333 | 0.1840 | Yes |
| 4 | PA2G4 |  |  | 137 | 0.329 | 0.2414 | Yes |
| 5 | CBX3 |  |  | 152 | 0.308 | 0.2939 | Yes |
| 6 | DCTPP1 |  |  | 172 | 0.296 | 0.3439 | Yes |
| 7 | HSPD1 |  |  | 176 | 0.294 | 0.3953 | Yes |
| 8 | HSPE1 |  |  | 211 | 0.276 | 0.4402 | Yes |
| 9 | TMEM97 |  |  | 331 | 0.223 | 0.4666 | Yes |
| 10 | NPM1 |  |  | 351 | 0.216 | 0.5026 | Yes |
| 11 | PLK1 |  |  | 364 | 0.213 | 0.5388 | Yes |
| 12 | NOP56 |  |  | 396 | 0.201 | 0.5709 | Yes |
| 13 | UNG |  |  | 398 | 0.200 | 0.6060 | Yes |
| 14 | PHB |  |  | 802 | 0.138 | 0.5872 | Yes |
| 15 | MRTO4 |  |  | 864 | 0.131 | 0.6038 | Yes |
| 16 | PLK4 |  |  | 986 | 0.121 | 0.6122 | Yes |
| 17 | GNL3 |  |  | 1084 | 0.112 | 0.6216 | Yes |
| 18 | NOLC1 |  |  | 1104 | 0.111 | 0.6391 | Yes |
| 19 | SRM |  |  | 1160 | 0.107 | 0.6520 | Yes |
| 20 | FARSA |  |  | 1217 | 0.103 | 0.6642 | Yes |
| 21 | EXOSC5 |  |  | 1376 | 0.093 | 0.6638 | Yes |
| 22 | TBRG4 |  |  | 1574 | 0.083 | 0.6574 | Yes |
| 23 | RRP9 |  |  | 1583 | 0.082 | 0.6710 | Yes |
| 24 | IMP4 |  |  | 1806 | 0.072 | 0.6600 | No |
| 25 | NIP7 |  |  | 1858 | 0.070 | 0.6669 | No |
| 26 | AIMP2 |  |  | 2087 | 0.061 | 0.6534 | No |
| 27 | TCOF1 |  |  | 2208 | 0.057 | 0.6506 | No |
| 28 | NDUFAF4 |  |  | 2383 | 0.051 | 0.6411 | No |
| 29 | MPHOSPH10 |  |  | 2398 | 0.051 | 0.6485 | No |
| 30 | NOC4L |  |  | 2407 | 0.051 | 0.6566 | No |
| 31 | WDR43 |  |  | 2556 | 0.046 | 0.6489 | No |
| 32 | LAS1L |  |  | 2698 | 0.042 | 0.6412 | No |
| 33 | PRMT3 |  |  | 2757 | 0.040 | 0.6420 | No |
| 34 | NOP16 |  |  | 2768 | 0.040 | 0.6480 | No |
| 35 | GRWD1 |  |  | 2793 | 0.039 | 0.6523 | No |
| 36 | PES1 |  |  | 2797 | 0.039 | 0.6588 | No |
| 37 | WDR74 |  |  | 2874 | 0.037 | 0.6572 | No |
| 38 | PPAN |  |  | 2947 | 0.035 | 0.6557 | No |
| 39 | BYSL |  |  | 3020 | 0.033 | 0.6539 | No |
| 40 | SORD |  |  | 3056 | 0.033 | 0.6559 | No |
| 41 | PPRC1 |  |  | 3804 | 0.019 | 0.5797 | No |
| 42 | MYC |  |  | 3842 | 0.018 | 0.5789 | No |
| 43 | RABEPK |  |  | 3961 | 0.016 | 0.5692 | No |
| 44 | PUS1 |  |  | 4015 | 0.015 | 0.5662 | No |
| 45 | UTP20 |  |  | 4703 | 0.004 | 0.4937 | No |
| 46 | MYBBP1A |  |  | 4769 | 0.003 | 0.4874 | No |
| 47 | RRP12 |  |  | 5129 | -0.002 | 0.4495 | No |
| 48 | SLC19A1 |  |  | 5319 | -0.005 | 0.4303 | No |
| 49 | TFB2M |  |  | 5662 | -0.011 | 0.3958 | No |
| 50 | DDX18 |  |  | 5826 | -0.014 | 0.3808 | No |
| 51 | SUPV3L1 |  |  | 6342 | -0.022 | 0.3299 | No |
Table: GSEA details [plain text format]

  

Fig 2: HALLMARK\_MYC\_TARGETS\_V2: Random ES distribution      
 Gene set null distribution of ES for **HALLMARK\_MYC\_TARGETS\_V2**
